# Portuguese Journal of Pulmonology and Brazilian Journal of Pulmonology-"Aquele Abraço" ("A Great Big Hug")

**DOI:** 10.1590/S1806-37132014000600001

**Published:** 2014

**Authors:** António Morais, Carlos Robalo Cordeiro

**Affiliations:** Portuguese Journal of Pulmonology, Lisbon, Portugal, Editor-in-Chief of the Portuguese Journal of Pulmonology, Lisbon, Portugal; Portuguese Society of Pulmonology, Lisbon, Portugal, President of the Portuguese Society of Pulmonology, Lisbon, Portugal

At this time when both journals-the Brazilian Journal of Pulmonology, an official publication of the Brazilian Thoracic Association, and the Portuguese Journal of Pulmonology (PJP), an official publication of the Portuguese Society of Pulmonology (PSP)-are in a phase of internationalization and growing recognition within the context of the group of publications on respiratory disease, both having been indexed and having received impact factors, we find it opportune to consider strengthening ties with those to whom we feel closer. In fact, the major objective of the PJP is to promote greater visibility and impact of the academic respiratory research performed in Portugal and to create the conditions for the PJP to be considered a relevant journal, in the sense that the various international research groups can consider it a reliable and interesting channel for publishing and disseminating their research.^(^ 1 ^,^ 2 ^)^ This objective is the reason for the changes we have made in the PJP, namely the increasing presence of the English language, in order to facilitate global access to its content. At the same time, it is our purpose to strengthen the relationship with journals published by associations with which we have closer relationships, preferably with Portuguese and Spanish language journals. Brazil has a key place here, for obvious reasons: we speak the same language; we have largely the same names and the same origins; we belong to some of the same international associations; and we cooperate closely within the Community of English Language Countries. In addition, within the respiratory community in Portugal, there is special interest in the research performed in Brazil and in Brazilian authors who stand out internationally, which translates to a significant number of Portuguese pulmonologists regularly consulting publications of Brazilian origin. Furthermore, the largest number of international articles published in the PJP is of Brazilian origin, with a total of 11 articles originating from Brazil being published in 2012-13.^(^ 3 ^)^ Although there has been a clear predominance of original articles on different topics in respiratory disease and by different Brazilian research groups, other types of articles, such as editorials, at the request of the editorial board, have been published.^(^ 4 ^)^ The highest number of logins to the PJP website also originates from Brazil.^(^ 3 ^)^

The history of the scientific publications on pulmonology in Portugal began in 1969, with the release of "Pulmonology-Portuguese Journal of Respiratory Diseases", edited and owned by the Lung Disease Clinic at the Lisbon School of Medicine, approximately five years before the institution of the Portuguese Society of Respiratory Diseases (PSRD), which was later designated PSP. That was a quarterly bilingual (Portuguese-English) journal run by distinguished Professor Thomé George Villar. A product of the career and international reputation of its director, the journal adopted a model inspired by the most prestigious international journals, namely including English, as well as Portuguese, as the official language of the journal, which reflects the notion that this would be essential to the visibility and impact of national publications. In 1978, the journal was replaced by one called "Thoracic Medicine", which was also issued on a quarterly basis and was under the same management. In 1980, the first issue of the official publication of the PSRD, designated "PSRD Archives", was released. The publication was run by another major figure of pulmonology in Portugal: Professor António José Robalo Cordeiro. Over more than 30 years, in the course of which the current name "Portuguese Journal of Pulmonology" was adopted, various editors and editorial boards made the decisions that allowed the PJP to attain its current leadership status among the medical publications in Portugal, which is surely a result of it being indexed for PubMed in 2003, it publishing its first issue in Portuguese and English in 2005, it being hosted onto the Elsevier platform as of 2010, and it having received its first impact factor in 2011. In fact, last July, the PJP received an impact factor from Thomson Reuters-ISI Web of Knowledge for the fourth time consecutively: more specifically, a value of 0.855. The PJP is currently published every two months, being open to the publication of original basic or clinical research articles, which will undergo rigorous assessment by the editorial board and via peer review, in accordance with the journal's regulations.

After years of regular contact between the editorial boards of the Brazilian Journal of Pulmonology and the PJP, as well as of their respective associations, the current board members have decided to cooperate more closely, which will result in the contents of each journal being made more actively and readily available to the members of the two associations, in addition to encouraging the production of collaborative papers by research groups in the two countries. This strategy will surely bring greater visibility and greater impact to the contents published in each journal, in addition to fostering synergistic collaboration between researchers in the two countries.

Paraphrasing the great Brazilian poet and singer Chico Buarque de Holanda, in a song written in honor of the Portuguese people at the time of the revolution of April of 1974 (the year of the founding of the PSRD, which originated the PSP), now in a different context, "sei* que há léguas a nos separar, tanto mar, tanto mar, sei também quanto é preciso navegar, navegar ...*" (literally, "I know there are leagues separating us, so much sea, so much sea, I also know how necessary it is to sail, to sail ..."), but this "*abraço*" ("hug") will surely be extraordinarily fruitful.
